# 

**Published:** 1906-02

**Authors:** 


*Sixty-first Year.*

VOL. LXI. FEBRUARY, 1906. No. 7

### BUFFALO MEDICAL JOURNAL

Established 1845 by AUSTIN FLINT, M. D.

EDITOR:

WILLIAM WARREN POTTER, M. D.

ASSISTANT EDITORS:

WILLIAM C. KRAUSS, M. D. NELSON W. WILSON, M. D.

ASSOCIATE EDITORS:

JAMES WRIGHT PUTNAM, M. D. ERNEST WENDE, M. D.
JOHN PARMENTER, M. D. JOHN A. MILLER, PH. D.
HARVEY R. GAYLORD, M. D. MAUD J. FRYE, M. D.

